# Supplementary material for: Tachykinin-related peptide signalling is important for the immune response of the mealworm beetle Tenebrio molitor L
Source: Front Immunol. 2026 Feb 5;17:1725225. doi: 10.3389/fimmu.2026.1725225 (PMC12916423; doi:10.3389/fimmu.2026.1725225)
Supplement: Supplementary file 1 [file DataSheet1.docx]

**Supplementary materials: Tachykinin-related peptide signalling is important for the immune response of the mealworm beetle *Tenebrio molitor* L.**

Konopińska N^1^., Walkowiak-Nowicka K.^1^, Nowicki G.^2^, Keshavarz M.^3^, Chowański Sz.^1^, Rolff J.^3,4^ and Urbański A.^1^

^1^Department of Animal Physiology and Developmental Biology, Faculty of Biology, Adam Mickiewicz University, Poznań, Poland

^2^genXone S.A., Złotniki, Poland

^3^Evolutionary Biology, Institute for Biology, Freie Universität Berlin, Berlin, Germany

^4^Berlin-Brandenburg Institute of Advanced Biodiversity Research (BBIB), Berlin, Germany





**Fig. S1.** Changes in the expression levels of genes encoding TRP precursor in the brain (A-C), ventral nerve cord (D-F) and fat body (G-I) of *T. molitor* after activation of the immune system. The immune response was elicited by injection of *Escherichia coli* K16 (Ec), peptidoglycan of *Staphylococcus aureus* (PG), or Spätzle-like protein. Control – individuals injected with physiological saline. Due to the dynamic nature of the immune response, samples were collected 3, 6 and 24 hours after immune system activation. The values are the means ± SDs. Asterisks indicate statistically significant changes compared to those in control individuals; **p*≤0.05.





**Fig. S2.** Changes in the expression levels of genes encoding TRP receptor (*TRPR*) in the brain (A-C), ventral nerve cord (D-F), fat body (G-I) and haemocytes (J-L) of *T. molitor* after activation of the immune system. The immune response was elicited by injection of *Escherichia coli* K16 (Ec), peptidoglycan of *Staphylococcus aureus* (PG), or Spätzle-like protein. Control – individuals injected with physiological saline. Due to the dynamic nature of the immune response, samples were collected 3, 6 and 24 hours after immune system activation. The values are the means ± SDs. Asterisks indicate statistically significant changes compared to those in control individuals; **p*≤0.05, ***p*≤0.01.





**Fig. S3.** Changes in the expression levels of immune-related genes (*Toll* (A-C), *Relish* (D-F), *Domeless* (G-I), *Attacin 2* (J-L), *Tenecin 3* (M-O)) in the fat body of *T. molitor* after activation of the immune system. The immune response was elicited by injection of *Escherichia coli* K16 (Ec), peptidoglycan of *Staphylococcus aureus* (PG), or Spätzle-like protein. Control – individuals injected with physiological saline. Due to the dynamic nature of the immune response, samples were collected 3, 6 and 24 hours after immune system activation. The values are the means ± SDs. Asterisks indicate statistically significant changes compared to those in control individuals; **p*≤0.05, ***p*≤0.01, ****p*≤0.001, **** *p*≤0.0001.


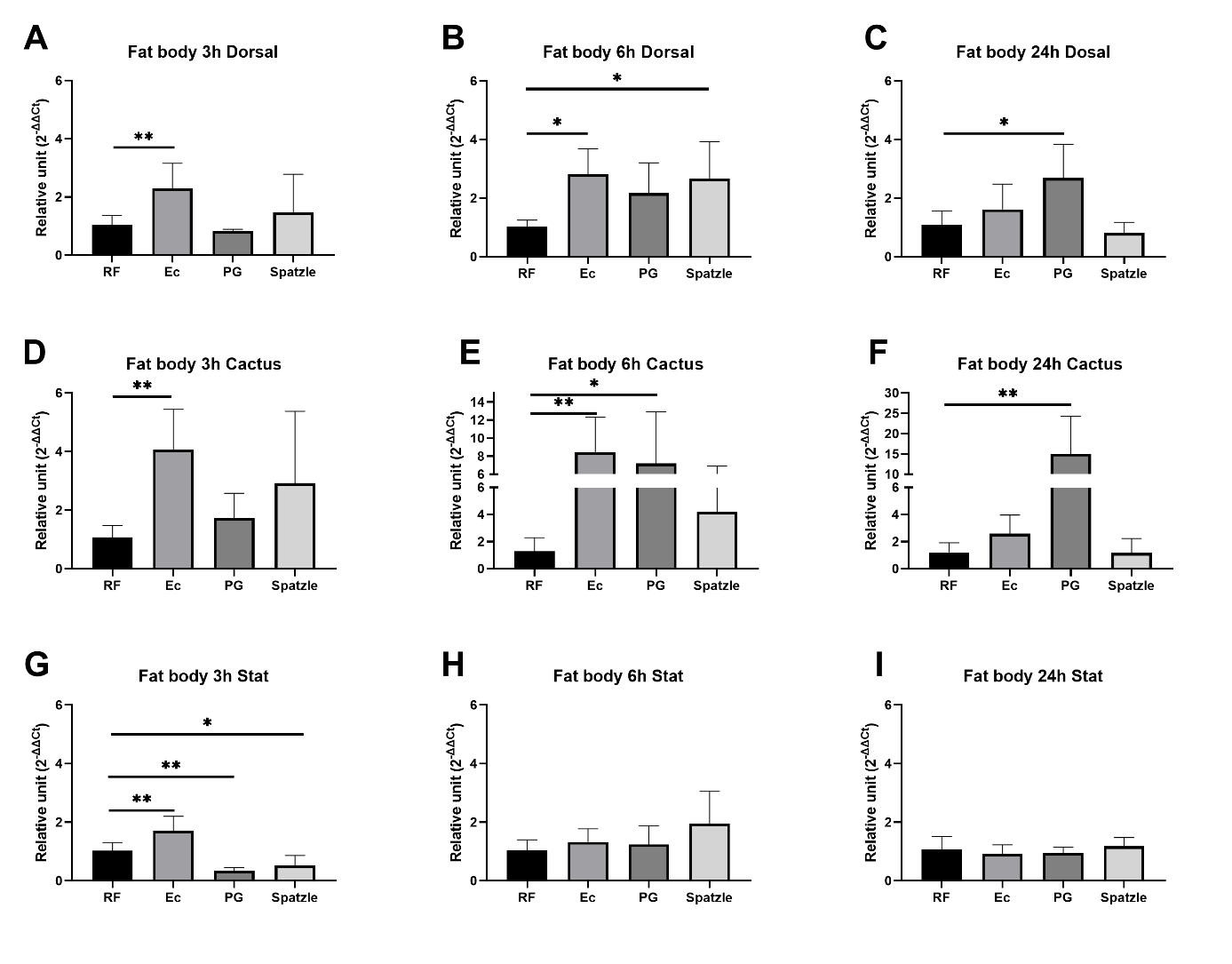


**Fig. S4.** Changes in the expression levels of immune-related genes (*Dorsal* (A-C), *Cactus* (D-F), *Stat92E* (Stat (G-I)) in the fat body of *T. molitor* after activation of the immune system. The immune response was elicited by injection of *Escherichia coli* K16 (Ec), peptidoglycan of *Staphylococcus aureus* (PG), or Spätzle-like protein. Control – individuals injected with physiological saline. Due to the dynamic nature of the immune response, samples were collected 3, 6 and 24 hours after immune system activation. The values are the means ± SDs. Asterisks indicate statistically significant changes compared to those in control individuals; **p*≤0.05, ***p*≤0.01.





**Fig. S5.** Changes in the expression levels of immune-related genes (*Toll* (A-C), *Relish* (D-F), *Domeless* (G-I), *Attacin 2* (J-L), *Tenecin 3* (M-O)) in the haemocytes of *T. molitor* after activation of the immune system. The immune response was elicited by injection of *Escherichia coli* K16 (Ec), peptidoglycan of *Staphylococcus aureus* (PG), or Spätzle-like protein. Control – individuals injected with physiological saline. Due to the dynamic nature of the immune response, samples were collected 3, 6 and 24 hours after immune system activation. The values are the means ± SDs. Asterisks indicate statistically significant changes compared to those in control individuals; **p*≤0.05, ***p*≤0.01, ****p*≤0.001, **** *p*≤0.0001.


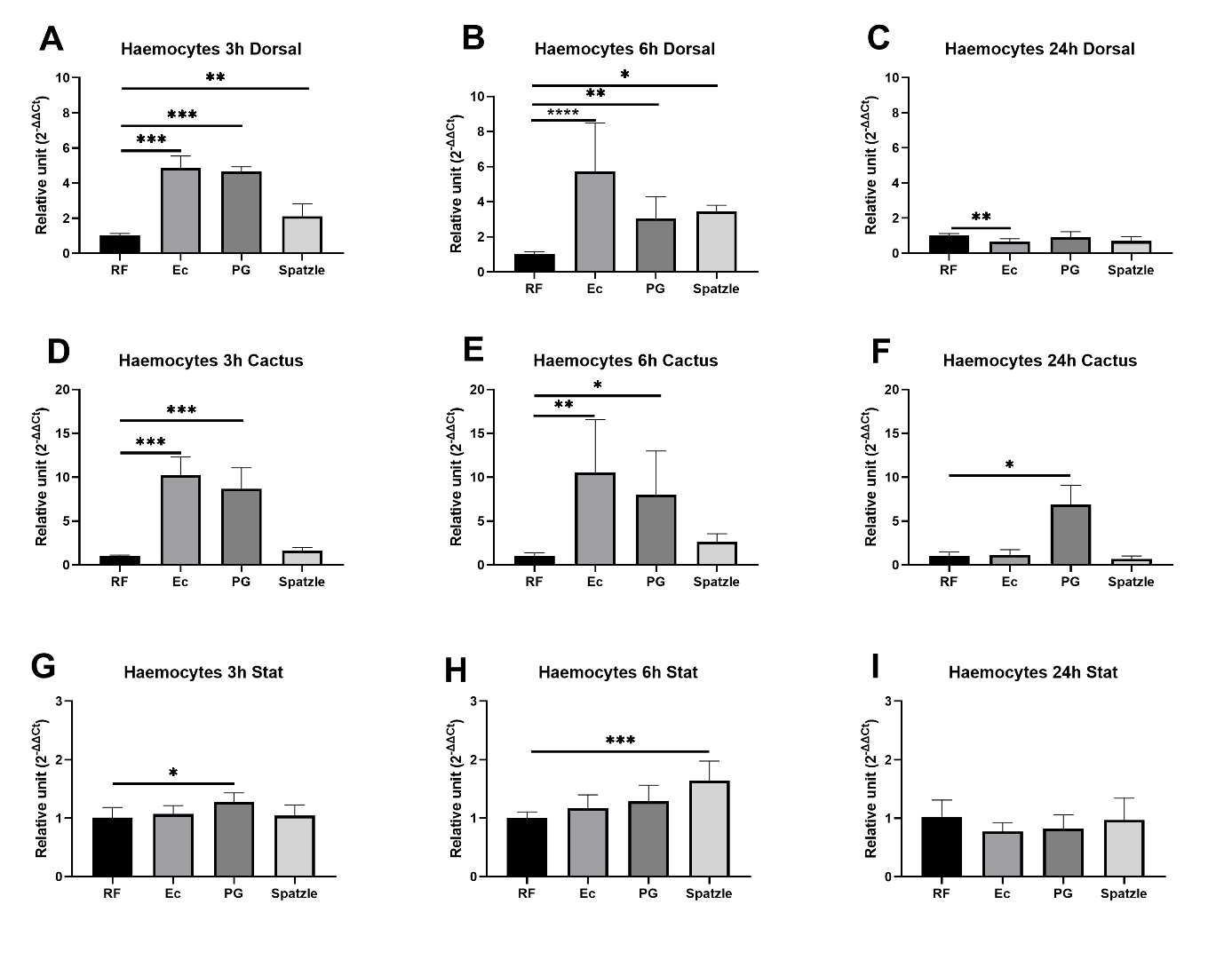


**Fig. S6.** Changes in the expression levels of immune-related genes (*Dorsal* (A-C), *Cactus* (D-F), *Stat92E* (Stat (G-I)) in the haemocytes of *T. molitor* after activation of the immune system. The immune response was elicited by injection of *Escherichia coli* K16 (Ec), peptidoglycan of *Staphylococcus aureus* (PG), or Spätzle-like protein. Control – individuals injected with physiological saline. Due to the dynamic nature of the immune response, samples were collected 3, 6 and 24 hours after immune system activation. The values are the means ± SDs. Asterisks indicate statistically significant changes compared to those in control individuals; **p*≤0.05, ***p*≤0.01, ****p*≤0.001, **** *p*≤0.0001.





**Fig. S7.** Changes in the expression levels of immune-related genes (*Toll* (A-C), *Relish* (D-F), *Domeless* (G-I), *Attacin 2* (J-L), *Tenecin 3* (M-O)) in the fat body of *T. molitor* after application of physiological saline (control), Tenmo-TRP-7 (TRP) at a concentration of 10^-5^ M, Spantide II at a concentration of 10^-3^ M, and a mixture of Tenmo-TRP-7 and Spantide II. Also after injection of dsRNA targeted genes encoding lysozyme in *Galleria mellonella* (*LysGm*, Control) or genes related to TRP signalling (genes for TRP precursor (dsRNA *TRP*) or receptor (dsRNA *TRPR*). In addition, the double-knockdown of *TRP* and *TRPR* was analyzed. Control for the double-knockdown experiment (Control 2KD) was the injection of the double dose of dsRNA-targeted *LysGm*. The values are the means ± SDs. Asterisks indicate statistically significant changes compared to those in control individuals; **p*≤0.05, ***p*≤0.01, ****p*≤0.001, **** *p*≤0.0001.


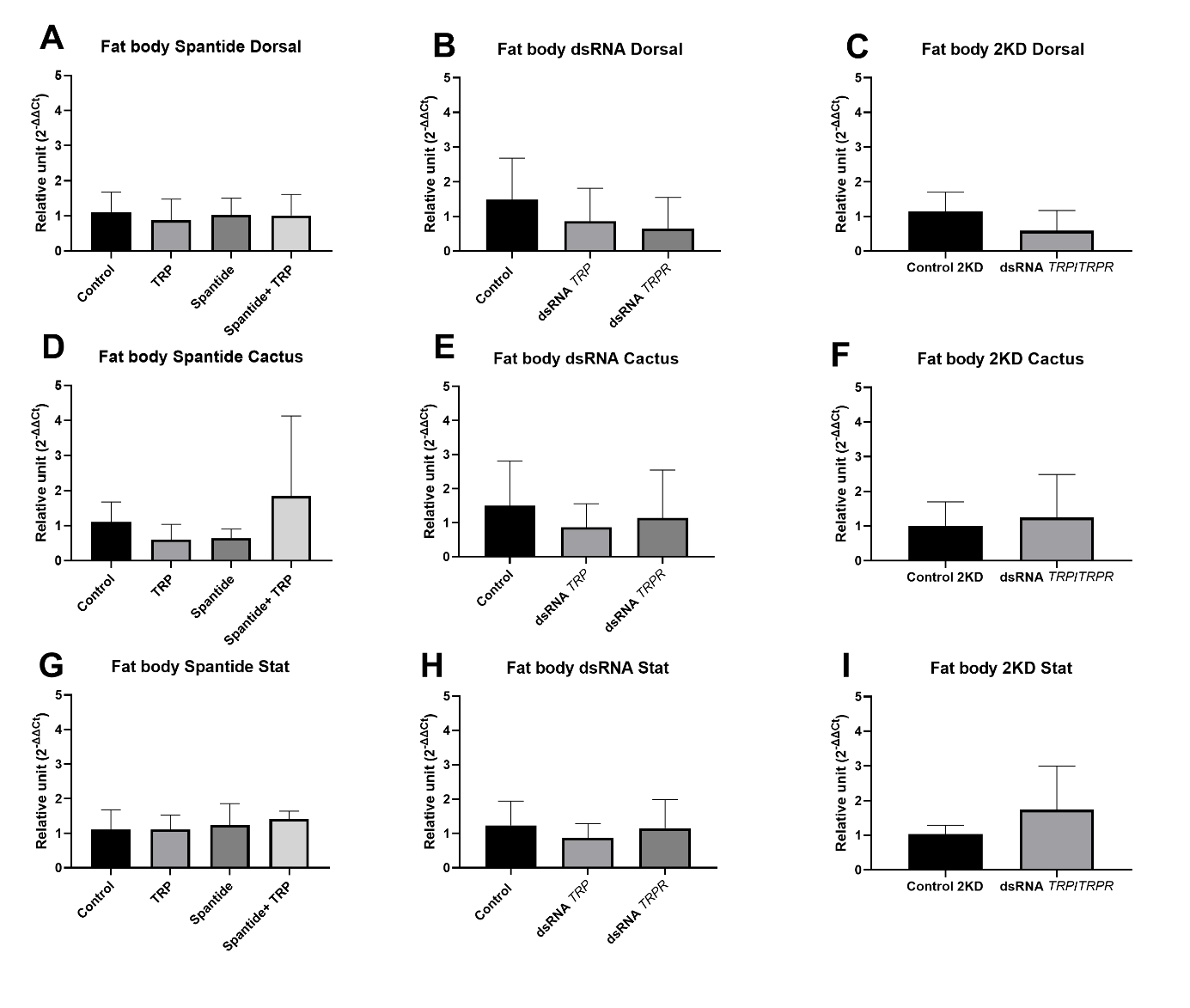


**Fig. S8.** Changes in the expression levels of immune-related genes (*Dorsal* (A-C), *Cactus* (D-F), *Stat92E* (Stat (G-I)) in the fat body of *T. molitor* after application of physiological saline (control), Tenmo-TRP-7 (TRP) at a concentration of 10^-5^ M, Spantide II at a concentration of 10^-3^ M, and a mixture of Tenmo-TRP-7 and Spantide II. Also after injection of dsRNA targeted genes encoding lysozyme in *Galleria mellonella* (*LysGm*, Control) or genes related to TRP signalling (genes for TRP precursor (dsRNA *TRP*) or receptor (dsRNA *TRPR*). In addition, the double-knockdown of *TRP* and *TRPR* was analyzed. Control for the double-knockdown experiment (Control 2KD) was the injection of the double dose of dsRNA-targeted *LysGm*. The values are the means ± SDs. Asterisks indicate statistically significant changes compared to those in control individuals.





**Fig. S9.** Changes in the expression levels of immune-related genes (*Toll* (A-C), *Relish* (D-F), *Domeless* (G-I), *Attacin* 2 (J-L), *Tenecin* 3 (M-O)) in the haemocytes of *T. molitor* after application of physiological saline (control), Tenmo-TRP-7 (TRP) at a concentration of 10^-5^ M, Spantide II at a concentration of 10^-3^ M, and a mixture of Tenmo-TRP-7 and Spantide II. Also after injection of dsRNA targeted genes encoding lysozyme in *Galleria mellonella* (*LysGm*, Control) or genes related to TRP signalling (genes for TRP precursor (dsRNA *TRP*) or receptor (dsRNA *TRPR*). In addition, the double-knockdown of *TRP* and *TRPR* was analyzed. Control for the double-knockdown experiment (Control 2KD) was the injection of the double dose of dsRNA-targeted *LysGm*. The values are the means ± SDs. Asterisks indicate statistically significant changes compared to those in control individuals; **p*≤0.05, ***p*≤0.01, ****p*≤0.001, **** *p*≤0.0001.


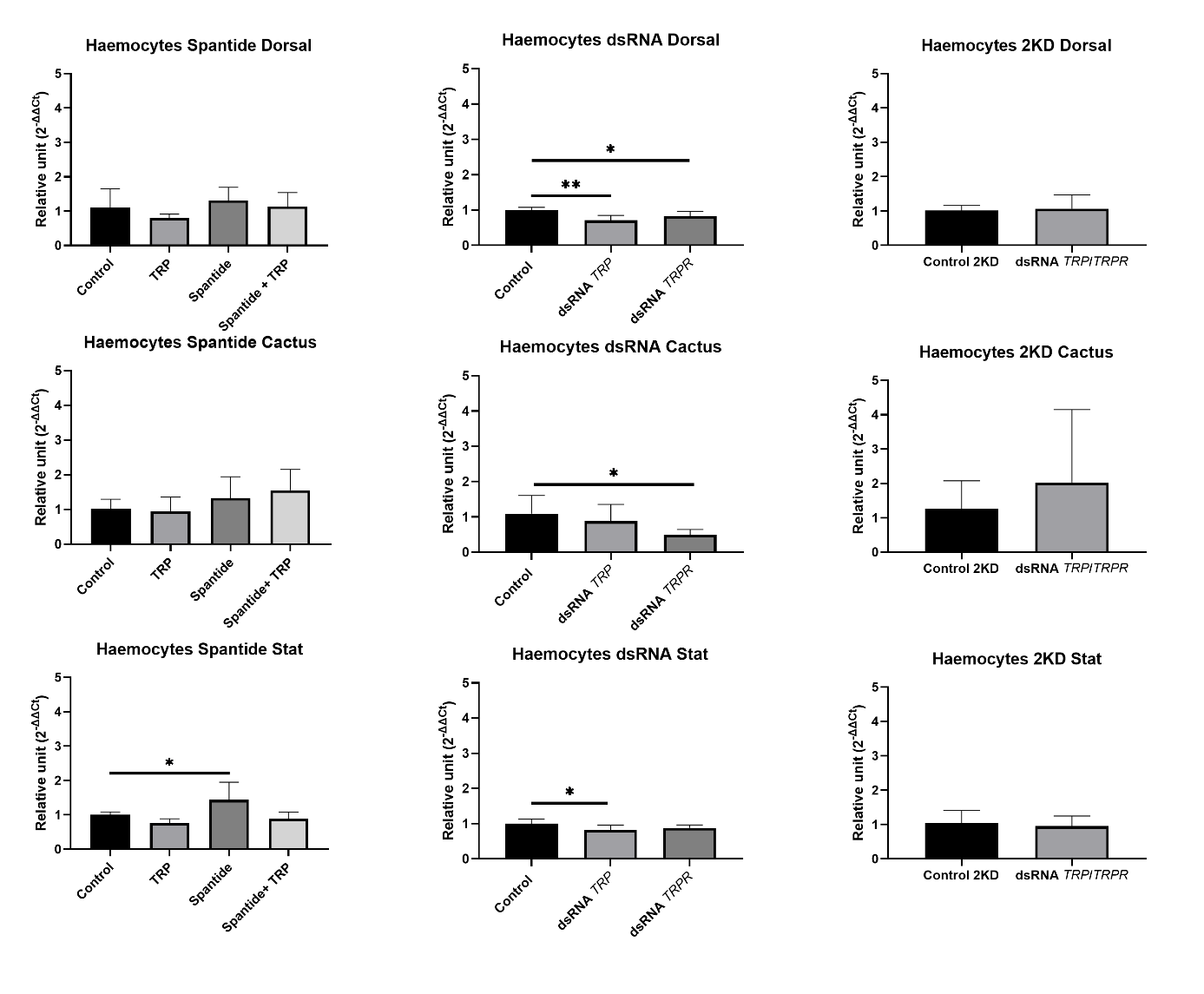


**Fig. S10.** Changes in the expression levels of immune-related genes (*Dorsal* (A-C), *Cactus* (D-F), *Stat92E* (Stat (G-I)) in the haemocytes of *T. molitor* after application of physiological saline (control), Tenmo-TRP-7 (TRP) at a concentration of 10^-5^ M, Spantide II at a concentration of 10^-3^ M, and a mixture of Tenmo-TRP-7 and Spantide II. Also after injection of dsRNA targeted genes encoding lysozyme in *Galleria mellonella* (*LysGm*, Control) or genes related to TRP signalling (genes for TRP precursor (dsRNA *TRP*) or receptor (dsRNA *TRPR*). In addition, the double-knockdown of *TRP* and *TRPR* was analyzed. Control for the double-knockdown experiment (Control 2KD) was the injection of the double dose of dsRNA-targeted *LysGm*. The values are the means ± SDs. Asterisks indicate statistically significant changes compared to those in control individuals; **p*≤0.05, ***p*≤0.01.


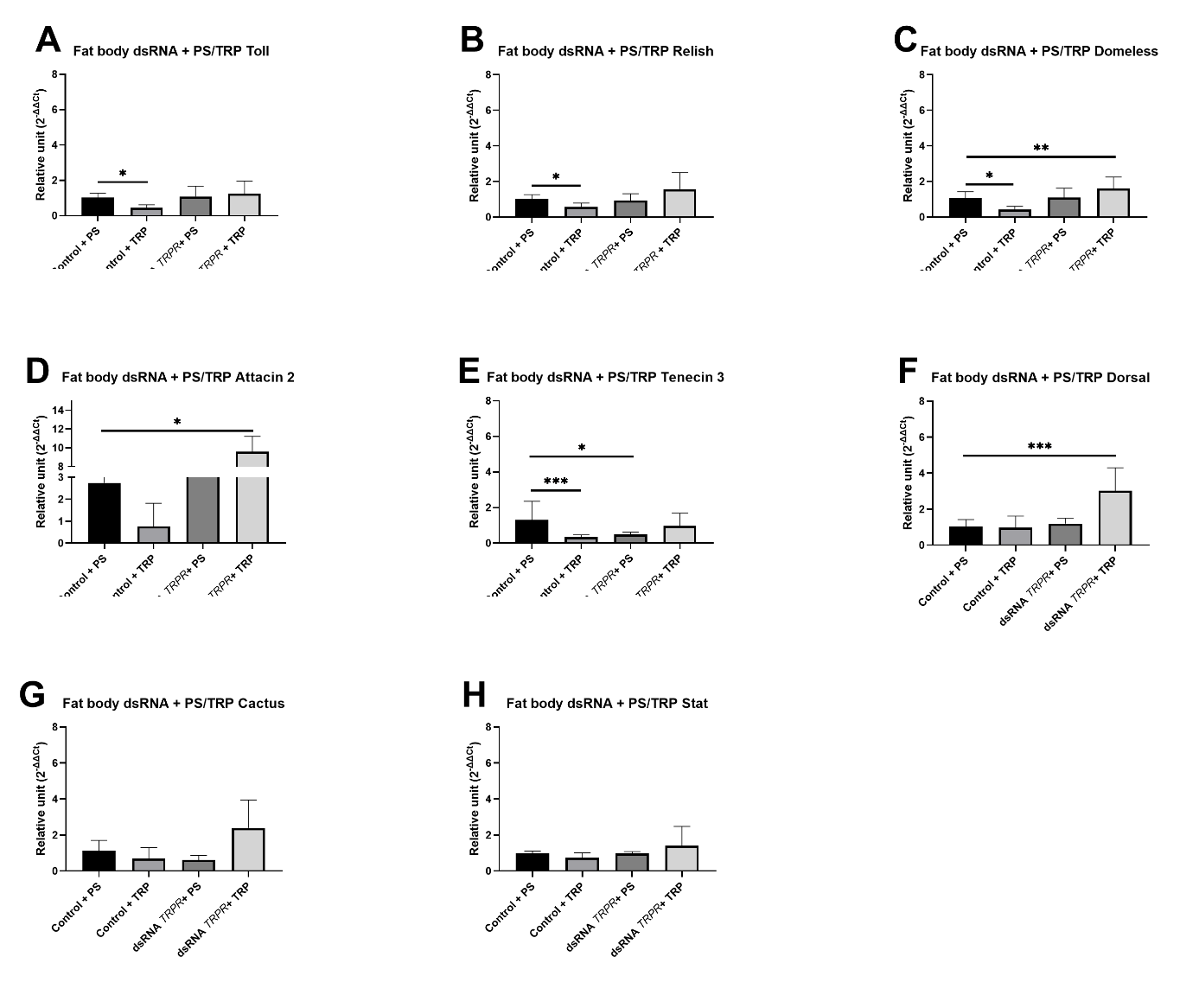


**Fig. S11.** Changes in the expression levels of immune-related genes (*Toll* (A), *Relish* (B), *Domeless* (C), *Attacin* 2 (D), *Tenecin* 3 (E), *Dorsal* (F), *Cactus* (G) and *Stat92E* (Stat, H)) in the fat body of *T. molitor* after application of dsRNA targeted genes encoding lysozyme in *Galleria mellonella* (*LysGm*, Control) and with physiological saline or Tenmo-TRP-7 (TRP) at a concentration of 10^-5^ M. In addition effect of injection of dsRNA targeted TRP receptor (dsRNA *TRPR*) and physiological saline or Tenmo-TRP-7 (TRP) at a concentration of 10^-5^ M was analyzed. The values are the means ± SDs. Asterisks indicate statistically significant changes compared to those in control individuals; ****p*≤0.001.


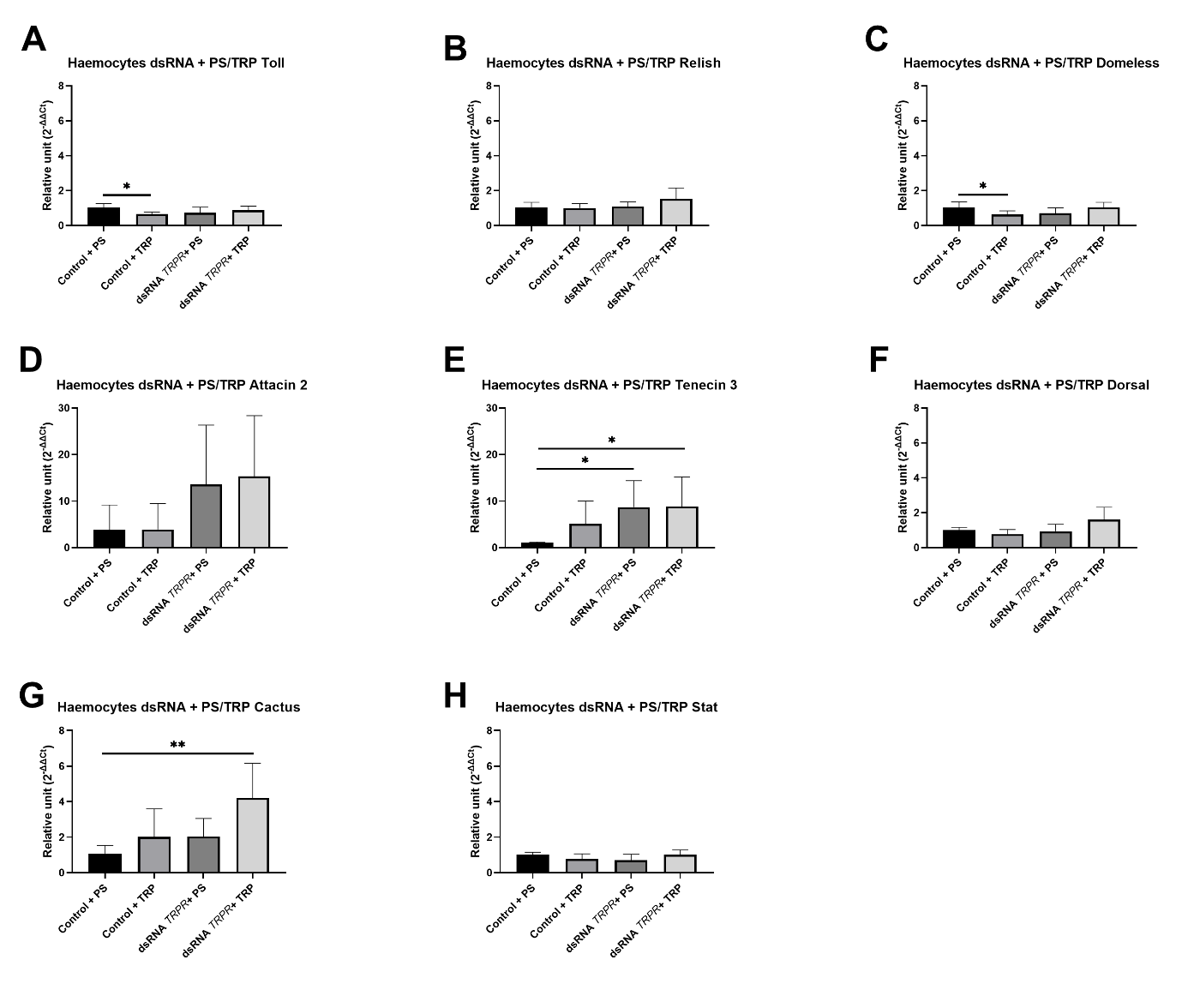


**Fig. S12.** Changes in the expression levels of immune-related genes (*Toll* (A), *Relish* (B), *Domeless* (C), *Attacin* 2 (D), *Tenecin* 3 (E), *Dorsal* (F), *Cactus* (G) and *Stat92E* (Stat, H)) in the haemocytes of *T. molitor* after application of dsRNA targeted genes encoding lysozyme in *Galleria mellonella* (*LysGm*, Control) and with physiological saline or Tenmo-TRP-7 (TRP) at a concentration of 10^-5^ M. In addition effect of injection of dsRNA targeted TRP receptor (dsRNA *TRPR*) and physiological saline or Tenmo-TRP-7 (TRP) at a concentration of 10^-5^ M was analyzed. The values are the means ± SDs. Asterisks indicate statistically significant changes compared to those in control individuals; **p*≤0.05, ***p*≤0.01.


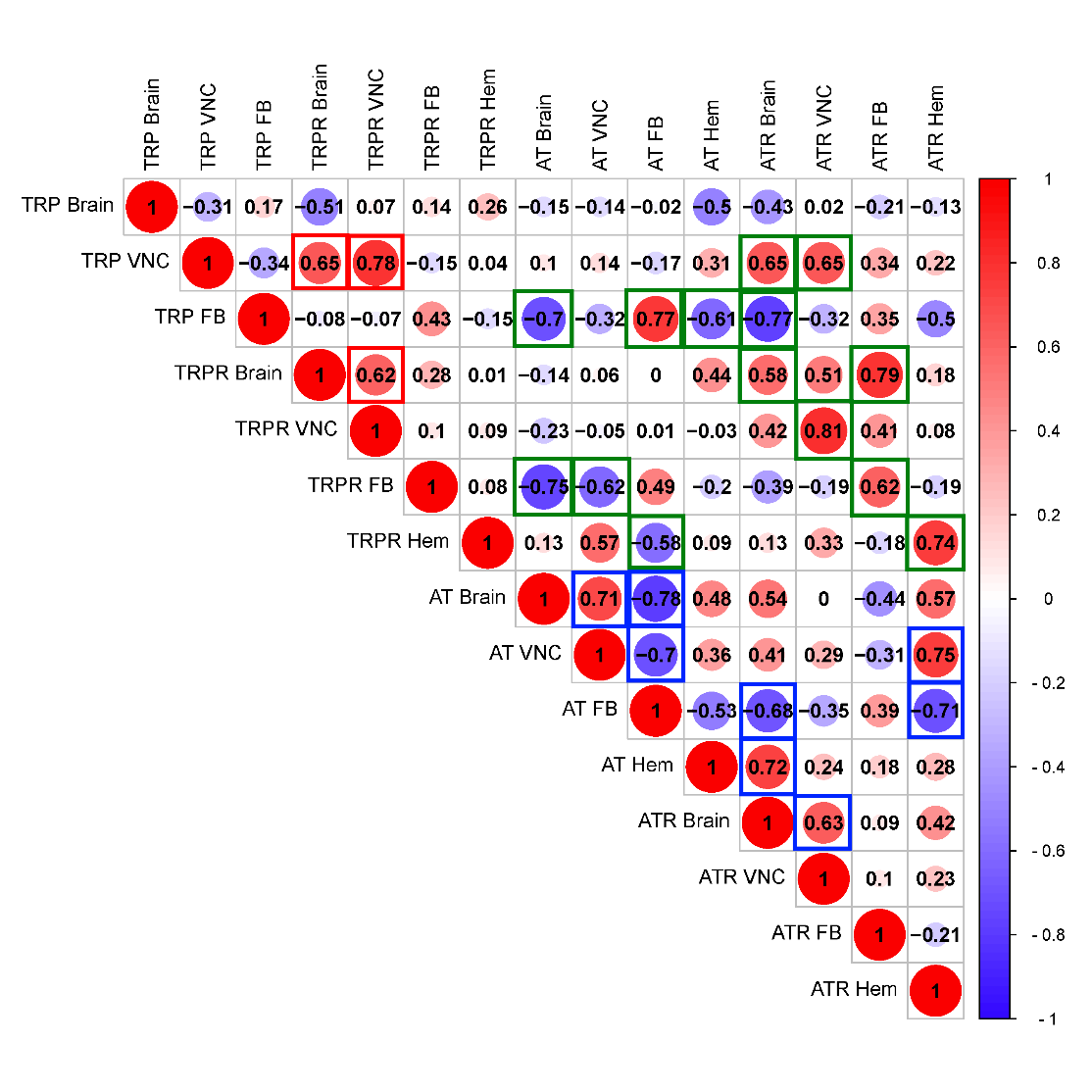


**Fig. S13.** Correlation of expression level of *TRP* and *TRPR* in different tissues/cells during activation of *Tenebrio* immune system with the expression level of genes encoding allatotropin precursor (AT) and receptor (ATR). Red squares – significant correlations associated with the TRP system presented in this article. Blue squares – significant correlations related to AT system (data previously presented by Konopińska et al. (2024)). Green squares – significant correlations between TRP and AT systems. VNC – ventral nerve cord; FB – fat body; Hem – haemocytes. To estimate the correlation of the data, the Pearson correlation coefficient method was used. The matrix was generated using SRplot software (https://www.bioinformatics.com.cn/srplot). Dot size – the level of the r value. The r value is presented in the middle of the dot. Different colors indicate different *r* values. Red shading indicates positive correlations (r > 0) and blue shading indicates negative correlations (r < 0). Red squares indicate statistically significant correlations (*p* ≤ 0.05).

**Tab. S1.** Sequences of primers used in the PCR analysis.

| **Name** | **Forward primer** | **Reverse primer** | **References** |
| --- | --- | --- | --- |
| **ds*LysGm*** | TAATACGACTCACTATAGGGAGAGCAAGCCGAATAAAAATGGA | TAATACGACTCACTATAGGGAGATATCTGGCAGCGGCTTATTT | Zanchi et al. 2017 |
| **ds*TRP*** | TAATACGACTCACTATAGCGGAATGAGGGGCAAGAAGT | TAATACGACTCACTATAGCGCGCCATCATTTTCAGTCA | This study |
| **ds*TRPR*** | TAATACGACTCACTATAGCGTCCGGGGGCTCTAACTAGA | TAATACGACTCACTATAGCGGGAGGTTGGAGTAGCAAG | This study |
| ***TmRpL13a*** | TCGTCGTGAGATGCGAACAA | CTGCTTCCCACGTTCTGTCT | Jacobs et al. 2017 |
| ***Tenmo-TRP*** | ATACGACAAACGAGCACCGT | GGGCATTTTCTTCCCCCTCA | This study |
| ***Tenmo-TRPR*** | TTATCCAGAATGGCCCGACG | CATTTGCCTCTGCGTGCATT | Urbański et al. 2021 |
| ***Toll*** | TGCGTAGCAAACAGGTGGAT | TCGCGTAGCGGTAGTAGAGA | Jacobs et al. 2017 |
| ***Cactus*** | TTCGTGACATCAGTGCGCTA | CATGTAGTTGTCGCCGGACT | This study |
| ***Dorsal*** | ACCCTTCGCAAATCTGCATCT | GCACCTTCGCAGAGGTCATT | This study |
| ***Relish*** | AGCGTCAAGTTGGAGCAGAT | GTCCGGACCTCATCAAGTGT | Keshavarz et al. 2020 |
| ***Domeless*** | CGCAACGGTGAGCATGTAAG | AATCGGCAGTGTCGAAGGTT | Urbański et al. 2021 |
| ***Stat92E*** | TGTCTTGGTCACTCACGTCC | TGTACCCTTGCATCAGCTCG | This study |
| ***Tenecin 3*** | CATCACGACGGACATCTGGG | TAAATGTCCGCCTGGTTGGC | Jacobs et al. 2017 |
| ***Attacin 2*** | CGAAGCAGTTCCGTCCATCT | CTCCTCCACAGGTTCGCATT | Jacobs et al. 2017 |
